# The Role of The African Vaccine Regulatory Forum (AVAREF) in The Accelerated Clinical Evaluation of Ebola Vaccine Candidates During the Large West Africa Epidemic

**DOI:** 10.29245/2578-3009/2018/si.1111

**Published:** 2018-09-01

**Authors:** Bartholomew Dicky Akanmori, David Mukanga, Ahmed Bellah, Tieble Traore, Michael Ward, Richard Mihigo

**Affiliations:** 1 Immunization and Vaccines Development, Family and Reproductive Health Cluster, WHO Regional Office for Africa; 2 Regulatory Systems Strengthening, Essential Medicines and Health Products, WHO Headquarters

**Keywords:** Role, Vaccine, African Vaccine Regulatory Forum, Ebola, Clinical Evaluation

## Abstract

In emergency situations, clinical trials of new vaccines and therapies in resource-constrained settings place an additional burden on the limited resources of low and middle-income countries. The clinical trials of vaccines against Ebola Virus Disease (EVD) in Africa presented challenges on how to ensure there was enough capacity for ethics and regulatory reviews and oversight while still allowing for accelerating the clinical evaluations. Using the African Vaccine Regulatory Forum (AVAREF) platform WHO supported African countries to provide ethics and regulatory reviews and oversight, ensuring that these trials were completed in unprecedented shorter timelines than normal, that is, months instead of years. Pathways were defined, external expertise provided and appropriate review models implemented in the countries which hosted these critical studies.

This paper discusses the work around the clinical trials, the models of reviews and timelines for clinical trials and highlights the important lessons revealed. More investments are required to monitor safety during clinical trials, strengthen systems for licensure of new products and implement robust post-marketing surveillance, among other components for effective clinical trials before the next pandemic surfaces.

## Introduction

The outbreak of Ebola Virus Disease (EVD), which primarily affected three countries in West Africa but spread to several others^1^, led to global research and development (R&D) agenda, requiring accelerated clinical evaluation of several products, including vaccines, monoclonal antibodies and other biologicals, to address the catastrophe^2–5^. The initial rush to have better case detection, outbreak response and control measures, was soon followed later by the R&D, which was hurried to ensure that efficacy trials of therapies and vaccines could be completed before the decline of the epidemic.

With no licensed EVD treatment or vaccine, it was an imperative to rapidly take all products available and with demonstrated potential from preclinical development through clinical trials and into efficacy studies in the affected countries. The WHO played its roles in global coordination; developed and promoted international norms and standards, provided guidance and evidence-based policy recommendations for R&D^6^.

The organization convened all the relevant stakeholders in R&D, namely sponsors, ethics committees, regulatory authorities, funding agencies, clinical investigators, research laboratories and data experts, working in concert to ensure that safe and efficacious products would be developed as quickly as possible. Through its leadership and coordination stakeholders agreed on clinical trial designs, ethics and regulatory pathways, data requirements, trial monitoring and oversight, trial consortia and capacity building requirements for these clinical trials.

The clinical evaluation of products in these countries whose health systems were already overwhelmed also placed an additional burden on systems for ethics reviews and regulatory approvals and oversight. Much of the work of the WHO involved consultations, consensus, building, promoting the public good, ensuring transparency, defining clear roles and responsibilities, supporting engagement and mobilization and reliance on the right expertise.

The R&D during the Ebola crisis was a departure from the normal R&D pathway for vaccines which typically takes several years, with sequential well defined, rarely overlapping steps, of preclinical development, clinical trials phases 1, 2 and 3, and culminating in product registration and introduction. Clinical trials are well designed, standardized and carefully executed experiments in humans, with the aim of determining the safety, efficacy, immunogenicity or mode of action of a new product. Ethics and regulatory reviews, as well as the regulatory authorization required for the importation and use of investigational new products, form an important part of product development, assuring participant safety and contributing to the reliability of data generated.

Several reviews have been published on research ethics in Africa^7,8^. All of these conclude that ethics and regulatory oversight for clinical trials in Africa are weak. The specific issues are the prolonged timelines for reviews of clinical trial applications, disparities in procedures and processes for reviews and authorizations of clinical trials and unclear roles and responsibilities for ethics committees and regulators. With several vaccine candidates in preclinical development and plans to take these into clinical trials in African countries, it became imperative to ensure that ethics and regulatory pathways are established, and these institutions were supported to conduct reviews, authorizations of clinical trials and to provide the required oversight.

The African Vaccine Regulatory Forum (AVAREF), the network of regulatory authorities and ethics committees in Africa served as a platform for the ethics and regulatory evaluations and oversight for the clinical trials of candidate vaccines against Ebola. The network prioritized regulatory oversight for these products by African National Regulatory Authorities (NRAs) with support from other regulatory agencies (including US Food and Drug Administration, European Medicines Agency, Health Canada). The African NRA established common understanding and agreement to review Clinical Trial Applications (CTAs) with the data available, undertook joint and assisted reviews of CTAs, and expedited reviews and decisions.

This manuscript describes how the AVAREF platform was used to fast-track all aspects of developing and delivering of effective and safe Ebola vaccines.

The paper discusses the accelerated clinical trials of candidate vaccines against EVD. It describes how this was achieved using AVAREF as a platform for the ethics and regulatory reviews and oversight. It also highlights how WHO utilized the AVAREF platform to ensure that these clinical trials of relatively complex and new products received the best reviews possible and that the trials were conducted in time to yield results for an informed decision on the use of vaccines in the outbreak.

## AVAREF, Platform For Ethics and Regulatory Support for R&D in Africa

The primary goal of setting up AVAREF in 2006 by WHO was to build capacity as quickly and effectively as possible to ensure that oversight could be provided for clinical trials in sub-Saharan Africa. AVAREF was set up at a time when evidence from a survey which was carried out by WHO in 2005 established that ethics and regulatory reviews and oversight of clinical trials of vaccines in the African region were weak^8^. The network uniquely brings together NRAs and ethics committees of the countries in the WHO African Region, promoting collaboration and information-sharing between them while ensuring that their roles and responsibilities are made much clearer, to minimize duplication of efforts in reviews of CTAs.

The network aimed to support the NRAs to take their responsibility of regulatory decision-making. It provides information on pipelines of product for clinical trials and target countries, promotes communication and collaboration between African NRAs and ethics committees and the expertise and advice of regulators from Europe and North America. Through the network, convergence towards harmonization of procedures and processes are also promoted.

AVAREF quickly established its innovative regulatory pathways for clinical trials, involving joint reviews and use of external experts identified by the WHO. Additionally, the network took an important step towards convergence through development and implementation of common guidelines for submission of clinical trial applications. These activities improved the quality of reviews and approvals of clinical trials in the African region. The collaboration among the members and with the strong participation of more stringent regulators has also improved product development^7, 9,10^.

## The Ethics and Regulatory Reviews of Clinical Trial Applications for Vaccines Against EVD

Recognizing the unique opportunity available, the WHO chose the AVAREF platform to support the ethics and regulatory reviews for the clinical trials of vaccines against Ebola during the emergency. By November 2014, there were few candidate vaccines under development. These candidate vaccines were new viral vectors. The viral vectors were human adenovirus type 26, a vesicular stomatitis virus also containing the genetic sequence for a glycoprotein of ZEBOV and a chimpanzee adenovirus type 3 also carrying ZEBOV glycoprotein sequence. Human data for these products were limited, especially data from Africans.. These candidate vaccines had only been tested in non-human primates. The lack of human safety data from any part of the world for these vaccines placed an additional challenge no the African regulators who were expected to authorize their use in relatively large phases 2 and 3 human clinical trials.

Consensus on the most appropriate pathways, the minimum data to consider for reviews, and mechanisms on how to ensure that oversight and assessment of the safety of clinical trial participants, especially of children and pregnant women was reached among the regulators and ethics committees. The AVAREF members also discussed with the sponsors the clinical trial designs of these studies, some of which were a departure from the standard placebo-controlled randomized trial designs, due to the disease, and pressure to use any products with potential as quickly as possible^11^.

There were various types of candidate vaccines against EVD available for clinical evaluation in the African region in 2014. These were viral vectors containing inserts of the glycoprotein of Zaire Ebola virus (ZEBOV) and a recombinant protein. The viral vectors were human adenovirus type 26, a vesicular stomatitis virus also containing the genetic sequence for a glycoprotein of ZEBOV and a chimpanzee adenovirus type 3 also carrying ZEBOV glycoprotein sequence. Human data for these products was limited especially data from Africans. The data are summarized in Table 1.

## Reviews of Clinical Trials and Timelines for Approvals

Three types of reviews were conducted during the period, individual reviews by ethics committees(ECs) and National Regulatory Authorities (NRAs), joint reviews convened by WHO for ECs and NRAs of target countries, assisted reviews convened by WHO, and assisted reviews without the involvement of WHO. Table 2 summarizes the trials, modes of reviews, timelines and status of trials. In general, the timelines for reviews were relatively shorter than for regular vaccine clinical trials which have taken place in the region. The expertise provided in the assisted review to the ethics committee and regulatory authority of Guinea for the phase III clinical trial protocol resulted relatively shorter timelines, contributing to an early commencement of the trial and its subsequent completion o before the outbreak ended (Henao-Restrepo, Longini et al. 2015. Lancet). The phase I clinical trials conducted in Kenya, Mali, and Gabon, were individually reviewed and took a matter of weeks for approvals to be obtained (Table 2).

The joint reviews of the clinical trial applications for the candidate vaccines against EVD, also highlighted the lack of clarity of the roles and responsibilities of ethics committees and regulators in the processes in countries of the region. During the reviews queries about the protocol raised by ethics committees were also similarly raised by the regulators and it soon became clear that roles and expertise were switched in some instances. The AVAREF platform brings together ethics committees and regulators, stressing the need for clarity in roles and responsibilities in order to minimize duplicationof efforts and to prolong review timelines. WHO provided guidance during the reviews ensuring that overlaps were kept to a minimum.

## Discussion

The international response to the EVD outbreak in West Africa tested the assumption that if clinical development is fast-tracked, new vaccines can be used to prevent an outbreak from becoming endemic or assuming epidemic proportions.. Less than one year later several EVD candidate vaccines were tested in clinical field trials in the African region. This fast-track development and conduct of field trials was partly due to the intensive work by ethics committees and regulatory bodies.

The geographic scope and urban nature of the outbreak of EVD demanded a global R&D response to deliver a reliable therapy or vaccine to prevent disease and to contain the outbreak and reduce mortality among the sick. Instead of vaccines being developed, clinically tested and licensed for use within the normal 5 to 10 years, vaccines against EVD were planned to be developed, tested and to be deployed in less than one year.

Ethics committees and regulators have a primary responsibility to ensure the protection of research subjects, generation of reliable data, which will result in the registration of the final product and its utilization by those who need the products the most. One of the challenges that affect reviews of clinical trials, in general, is the complexity of the product tested and the clinical trial design^12–14^. The two vaccines which have successfully undergone clinical trials in Africa and within the AVAREF network in the past have been recombinant protein-based products, such as the conjugate meningitis A vaccine and the RTS,S/AS01 malaria vaccine. The two vaccines were also tested in several earlier phases of clinical trials before the efficacy trials.

In contrast, the candidate vaccines against EVD were viral vectors, unfamiliar to the regulators and ethics committees and with little human data to guide them in decision-making. This challenge could only be overcome, by work and expertise sharing, information sharing with regulators where INDs were submitted and the use of external experts provided by WHO to assess the risk-benefit profile of the products. The AVAREF served as the ideal platform having been formed as a network for experience and information sharing, as well as involving external expertise and with confidentiality agreements to protect the intellectual property of sponsors and owners of these candidate vaccines.

Depending on the scope of clinical trials, the number of countries involved in a trial, availability of expertise and the involvement of WHO in the trial any of the models for reviews of the applications can be chosen. An assisted review is often appropriate where just one country is involved and expresses an interest in having more expertise, while a joint review serves as the best means for multicenter clinical trials. Smaller phase one trials in individual countries can often be undertaken by individual reviews that may not require the involvement of additional countries. For example, the small phase I clinical trials in Mali, Kenya, and Gabon were reviewed by the ECs and NRAs of the countries individually^15,16^.

The critical issue in the development of vaccines for use in an emergency is how to meet the very strict timelines so that adequate clinical and safety data can be obtained for the product to be registered or used as part of the outbreak response. Such a demand for shorter timelines puts pressure on ethics committees and regulators, particularly in LMICs where institutions are already severely under-resourced. The ECs and NRAs will have to find additional experts within a very short time and also to expedite the reviews using their limited resources.The success of the ethics and regulatory approvals is also evident in the relatively shorter timelines for reviews and approvals of clinical trials.

Safety is considered a vital endpoint of all phases of clinical trials and beyond, into the post-licensure or marketing phase. Remarkably all the countries agreed on what would be an acceptable risk-benefit level for approvals. The major risk considered were likelihood of the vectors replicating and beig passed on in women who may become pregnant and any possible health consequences. Although the EVD vaccine clinical trial applications were thoroughly reviewed, the acceptable risk-benefit level agreed to, before approvals were given, the monitoring of the trial sites to ensure compliance across all study sites was limited. One country conducted a pre-trial site audit consistent with their regulations. The countries could not carry out audits of the trial sites primarily because they lacked the capacity and expertise. Although training was planned to address this gap, it never took place as planned. Future support for ethics and regulatory oversight of R&D in an emergency should build into the blueprint a robust training and joint inspections of trial sites to ensure that there is compliance with approval conditions.

Successful product development demands that in addition to ethics and regulatory systems, other systems, resources, and facilities are firmly put in place. Research infrastructure, including field sites, surveillance systems, laboratory capacity, good governance, partners and funding agencies, country ownership, community engagement and appropriate communication systems, remain vital. Most African countries still lack the infrastructure for research in general, and few trial sites have the capacity to respond rapidly to emergencies by hosting clinical trials as required during the outbreak of EVD. The three countries which were most affected by EVD and whose health systems were stretched to the limit in responding to the outbreak, faced even more severe challenges on reviews of clinical trial applications.

Using the AVAREF platform was used to convene joint and assisted reviews of the countries that hosted the clinical trials, bringing together the reviewers, sponsors, and expertise that enabled the countries to make well-informed decisions^17^. The AVAREF platform supported R&D of candidate vaccines against EVD in Africa. But the platform fulfilled only a part of requirements for successful product development. Additional requirements including adequately resourced sites, clinical investigator teams, laboratory and data management capacities, all of which contribute to successful product evaluation must be in place to successfully accelerate R&D. More investments are needed to ensure that the region has adequately resourced clinical trial sites, laboratory capacity, funded-investigator teams in readiness, adequate surveillance which can also pick adverse events following immunization, ethics and regulatory capacity and standard operating procedures to address the next epidemic.

## Figures and Tables

**Table 1:** Summary of Candidate Vaccine Types Against Ebola Virus Disease Available as of December 2014. The Manufacturer or Sponsor, Type of Vaccine, Availability or Otherwise of Human Data Target Populations are also Presented.

Candidate	Type	Primate data	Human data availability in December 2014	Products containing vector/product already tested in Africa?	category
GSK Chimpanzee Adenovirus type 3- ZEBOV	Non-human Viral vector	available	limited	Yes, malaria vaccine	Adults and children
Merck/Newlink recombinant Vesicular Stomatitis Virus-ZEBOV	Non-human non-replicating Viral vector	available	unavailable	No	
J&J Human Adenovirus type 26-ZEBOV/MVA	Human viral vector	available	available	Yes	adults
Novavax	Recombinant protein (non-live)	Available?	available	No	

**Table 2:** Types of Clinical Trial Reviews used and Approval Timelines For Candidate Vaccines against Ebola Virus Disease.

Candidate vaccine	Clinical trial phase	Target countries	Mode of review	Timeline	Trial status with references
GSK ChAd 3	Phase I	Mali, UK	Individual	2 weeks	Completed^15^
	Phase II	Ghana, Nigeria, Cameroun, Mali, Senegal	Joint	3 months	ongoing
	Phase III	Liberia			Could not be completed
					
Merck/Newlink r-VSV-ZEBOV	I	UK, Germany, Switzerland, Gabon, Kenya	Individual	2-3 weeks	Completed^18^
					
					
	III	Guinea	Assisted	2 weeks	Completed^19^
	III	Sierra Leone			Completed^20^
J&J Ad26-ZEBOV/MVA	I	*Ghana, Kenya, Uganda, Uganda, Tanzania	Joint	1-3 months	
	II				
	III	Sierra Leone			Could not be started

* Application was withdrawn because product shelf-life expired before approval.
